# Optimizing Temperature Setting for Decomposition Furnace Based on Attention Mechanism and Neural Networks

**DOI:** 10.3390/s23249754

**Published:** 2023-12-11

**Authors:** Shangkun Liu, Wei Shen, Chase Q. Wu, Xukang Lyu

**Affiliations:** 1 School of Computer Science and Technology, Zhejiang Sci-Tech University, Hangzhou 310018, China; 202220601006@mails.zstu.edu.cn; 2 Department of Data Science, New Jersey Institute of Technology, Newark, NJ 07102, USA; 3 Zhejiang New Rise Digital Technology Co., Ltd., Hangzhou 311899, China; clu@newrisedt.com

**Keywords:** CNN, LSTM, attention mechanism, LASSO, optimal setting, sensor

## Abstract

The temperature setting for a decomposition furnace is of great importance for maintaining the normal operation of the furnace and other equipment in a cement plant and ensuring the output of high-quality cement products. Based on the principles of deep convolutional neural networks (CNNs), long short-term memory networks (LSTMs), and attention mechanisms, we propose a CNN-LSTM-A model to optimize the temperature settings for a decomposition furnace. The proposed model combines the features selected by Least Absolute Shrinkage and Selection Operator (Lasso) with others suggested by domain experts as inputs, and uses CNN to mine spatial features, LSTM to extract time series information, and an attention mechanism to optimize weights. We deploy sensors to collect production measurements at a real-life cement factory for experimentation and investigate the impact of hyperparameter changes on the performance of the proposed model. Experimental results show that CNN-LSTM-A achieves a superior performance in terms of prediction accuracy over existing models such as the basic LSTM model, deep-convolution-based LSTM model, and attention-mechanism-based LSTM model. The proposed model has potentials for wide deployment in cement plants to automate and optimize the operation of decomposition furnaces.

## 1. Introduction

As an indispensable piece of equipment in the new dry cement sintering system [1], decomposition furnaces provide various functions such as pulverized coal combustion, gas–solid heat exchange, and carbonate decomposition [2]. The furnace temperature is an important index to judge the normal operation of calciner. If the furnace temperature is too low, it could not generate the heat required for carbonate decomposition, hence resulting in an insufficient decomposition rate [3] and low product quality. In this case, the rotary kiln has to undertake more role in decomposition, which increases the burden of the kiln system. On the other hand, if the furnace temperature is too high, coal consumption increases, resulting in the waste of resources and the increase in cost. Furthermore, a large difference in the temperature between the inside and outside of the furnace causes the liquefied raw meal to stick to the equipment when it comes into contact with the outlet low temperature, which causes the crust problem [4] and affects the service life of the equipment. Therefore, it is of great practical importance to optimize the temperature setting of the decomposition furnace to control the furnace temperature change and achieve a decomposition rate that meets the technical requirement of cement production.

Several previous efforts have been made to optimize the temperature setting of decomposition furnaces. Li [5] proposed a method based on case reasoning, which establishes a knowledge base of cases through experiences, and then provides a value setting according to certain matching rules. Zhao [6] proposed a rule-based reasoning method and improved Li’s work by adding suggestions on the identification and treatment of abnormal working conditions. However, the principles of these methods are still partially based on operating experiences, and cannot fully reflect the operator’s judgement on the working condition. In addition, Zhao [7] proposed a support vector regression algorithm optimized using cuckoo search (CS-SVR) to establish a prediction model of furnace temperature, and adjusted temperature through expert rules, which still suffer from the above limitations.

All of these methods are largely dependent on the operator’s operating experiences, and hence face the following challenges: (1) Even the most skilled operator may make mistakes in their judgement of working conditions, which may lead to frequent adjustments of optimal settings for correction, resulting in a waste of resources. (2) Factories are generally equipped with multiple operators, and different operators may come to different conclusions for the same working condition due to different cognition and operating habits. When handing over the job duty, the value setting could vary because of different cognitions, which would also cause a resource waste. Therefore, it is particularly important to automate and optimize temperature setting to address these issues.

Optimal temperature is a time-dependent variable. Since there are a large number of dimensions of working conditions, it is difficult to directly establish a model with both time series information and working condition characteristics. Neural networks provide a promising solution to this problem.

The Long Short-Term Memory (LSTM) model is a deep learning model specially designed for time series prediction [8]. It has been widely used in speech recognition [9,10], text recognition [11,12], and industrial fields [2,13,14,15]. However, the LSTM model has its own limitations when dealing with long time series [16,17]. Recent studies have shown that its long-term information processing ability is still a bottleneck [18]. Therefore, it remains a key challenge to establish a time-series model that can mine and memorize complex dependencies.

In [19,20], the authors discussed how to combine attention mechanism with LSTM to improve the accuracy of LSTM in predicting long-term time series. In addition, the information mining of multi-dimensional working condition characteristics also has an important influence on the performance of a prediction model. The LSTM hybrid model based on convolutional network can help solve this problem, as CNN enhances LSTM’s ability to store and learn nonlinear working condition characteristics [21]. After information mining by CNN, it is helpful to capture relevant data. This idea has been used in many problems, such as stock prediction [22,23], gold price prediction [24], blood sugar level prediction [25], food testing [26], etc.

Based on the principles of deep convolutional neural networks (CNNs), long short-term memory networks (LSTMs), and the attention mechanism, we propose a CNN-LSTM-A model to optimize the temperature setting for a decomposition furnace. The proposed model combines the features selected by Least Absolute Shrinkage and Selection Operator (Lasso) with others suggested by domain experts as inputs, and uses CNN to mine spatial features, LSTM to extract time series information, and an attention mechanism to optimize weights. We deploy sensors to collect production measurements at a real-life cement factory for experimentation and investigate the impact of hyperparameter changes on the performance of the proposed model. Experimental results show that CNN-LSTM-A achieves a superior performance in terms of prediction accuracy over existing models such as the basic LSTM model, the deep-convolution-based LSTM model, and the attention-mechanism-based LSTM model. The proposed model has the potential for wide deployment in cement plants to automate and optimize the operation of decomposition furnaces.

The main contributions of our work are summarized as follows:

1. We deployed sensors to collect measurements at a production cement plant and used the collected data to evaluate the performance of our model. Due to the infrequent temperature setting, it was challenging to extract the time series information. To address this issue, we sliced the data and used linear interpolation and smoothing methods to fill in the missing data and handle abnormal data. The input features of the model were selected according to suggestions from experts and the LASSO feature selection method.
2. We proposed the CNN-LSTM-A model, combining the advantages of CNNs, LSTMs, and attention mechanisms to predict optimal setting values, and trained the model on the original dataset.
3. The performance of CNN-LSTM-A was compared with an LSTM, a CNN-LSTM, and an LSTM-A. The experimental results show that the CNN-LSTM-A achieves higher accuracy and adaptability than other methods.
4. We run real-time tests at a production factory and prove the practicality and efficacy of the proposed model for production use.

According to our survey of the state of the arts in this field [5,6,7], we believe that this paper presents pioneering work on the applications of neural networks to optimizing the temperature settings of decomposition furnace.

The rest of the paper is organized as follows. Section 2 presents the proposed method. In Section 3, we describe data collection, perform feature processing, and pre-process raw data. In Section 4, we design the mixed model structure, determine the evaluation metrics, train each model with different hyperparameters, and compare their performance. In Section 5, we summarize our work and results.

## 2. The Overall Framework of the Proposed Model

In this section, we introduce the physical process of furnace and present the overall framework of the proposed model to optimize the temperature settings for the decomposition furnace.

### 2.1. Decomposition Process

**Figure 1:**
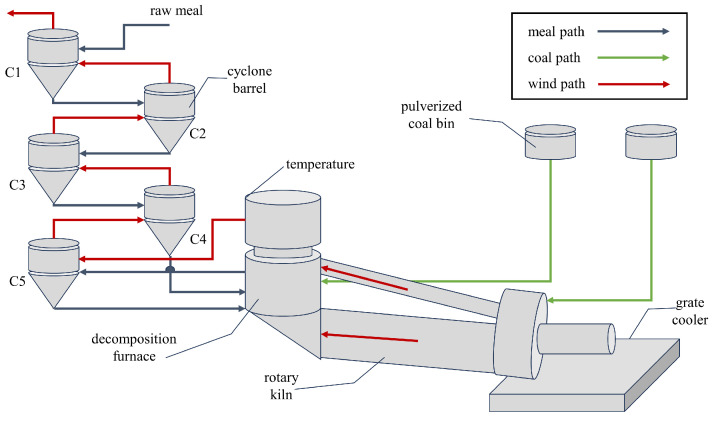
Firing system.

As shown in Figure 1, the firing system consists of five parts: preheater, decomposition furnace, rotary kiln, pulverized coal bin, and grate cooler.

Fuel enters from the coal bin into the decomposition furnace and rotary kiln. The fuel burned in the decomposition furnace releases gas with heat, which ascends from the C5 cyclone to the top C1 cyclone for discharge. Raw materials are entered from the C1 cyclone, undergoing heat exchange with rising gas under gravity, serving as a preheating function. After preheating in the C4 cyclone, the raw materials go into the decomposition furnace, where coal powder is ignited under the temperature of three-stage air, generating heat and gas. The materials are then moved to the C5 cyclone for collection before entering the rotary kiln for further processing.

The decomposition furnace is responsible for 60% of fuel combustion and over 90% of carbonate decomposition in the firing system, making it a crucial control factor in the process.

The system deploys various sensors, including pressure sensors to monitor raw material flow and temperature sensors to monitor gas temperature. Temperature sensors are mainly placed in the following locations:

1. Exit of cyclones: Each cyclone is equipped with a temperature sensor to detect the exit temperature, ensuring sufficient preheating.
2. Decomposition furnace: Three temperature sensors are installed to monitor changes in the exit, middle, and bottom temperatures. The most critical variable is the exit temperature, which reflects the internal operation of the equipment and serves as a vital reference for evaluating preheating and carbonate decomposition efficiency.

Based on the data collected by sensors, operators are able to judge the system’s current state based on their domain knowledge. If the outlet temperature of the decomposition furnace is too high or too low, the raw material decomposition rate would be insufficient and the final product quality would be compromised. In such cases, operators need to set the target temperature value to bring the system back to normal operation.

### 2.2. Problem Description

Temperature setting optimization for decomposition furnace is essentially a time series prediction problem. By rolling through a fixed time window of size *L*, we collect a data sequence $X={\left\{X_{1}^{t},\ldots ,X_{N}^{t}\right\}}_{t=1}^{L}$, which contains *N* variables, and each element $X_{i}^{t}$ represents the measurement of the i−th variable at time *t*. Given such a data sequence, we wish to predict the corresponding temperature $Y={\left\{Y^{t}\right\}}_{t=L+T}$ at future time *T*.

### 2.3. Model Structure

**Figure 2:**
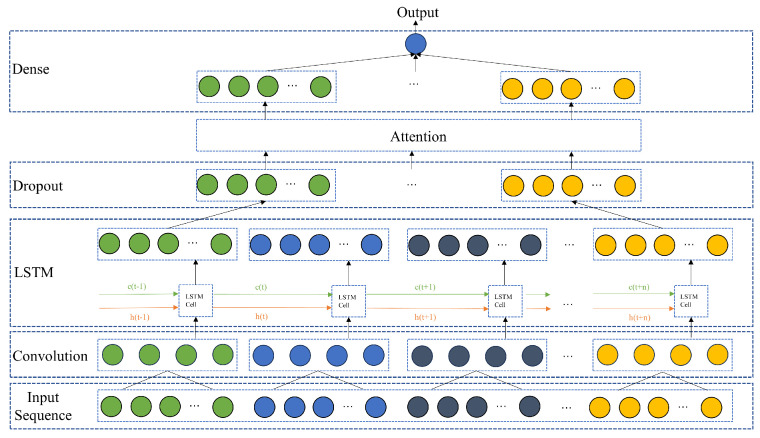
Structure of the proposed model.

The structure of the proposed model is shown in Figure 2. The convolution layer (CNN) is used to extract the spatial features of the multi-dimensional working condition input, and convolute the data vertically according to the set convolution Kernel step to extract abstract working condition features. The convolution process obtains the spatial distribution characteristics of sequence information and improves the depth of feature mining. The convolution network with weight sharing can greatly reduce the parameters of the network layer, which has a significant effect on improving the efficiency of model training.

The Long Short-Term Memory layer (LSTM) further mines the time series information from the convolution results, establishes the mapping relationship between multi-dimensional input parameters and optimal setting labels, predicts the output with a certain length of the input, and then merges the generated output with the input to predict the next output.

The Dropout layer discards some parameters to avoid overfitting, which reduces the complexity of the neural network and improves the efficiency of training. Following the work by Luong in [27], we use an attention mechanism to optimize the distribution of weights. The Dense layer compresses the output to the one-dimensional prediction value for optimal setting.

### 2.4. Optimization Process

**Figure 3:**
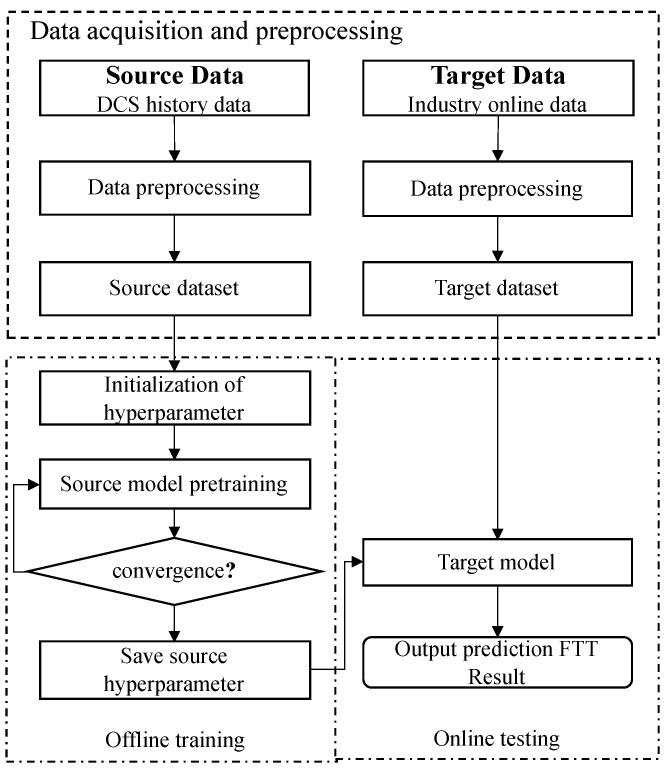
Optimal setting prediction model establishment process.

Based on the trained CNN-LSTM-A model, the prediction process for optimal temperature settings for the decomposition furnace is illustrated in Figure 3.

1. Data acquisition and pre-processing
  We deploy sensors to collect data at a cement plant as the source domain and the real-time monitoring data from the factory working condition monitoring system as the target domain. After data pre-processing, the source domain dataset and the target domain dataset are formed.
2. Offline training
  In offline model training, the source dataset is used for training, the Adam function is used to carry out back propagation to optimize the model, and the training parameters are saved when the model converges.
3. Online testing
  In the process of online testing, optimal setting is predicted according to the model parameters saved from training.

## 3. Data Processing

### 3.1. Experimental Dataset

In this paper, we use the data collected using the data acquisition system (DCS) of a cement plant in Jiangxi Province during a period from June to December in the second half of 2022 as the experimental dataset. We use $f_{1},\ldots ,f_{n}$ to denote the data dimension. Since the equipment failed to operate normally in the first half of June, there are many abnormal values in the dataset during this time window, which are abandoned in the experimental training.

### 3.2. Feature Selection

Since the *n* dimensional dataset far exceeds the appropriate dimension of deep learning input, and there are a large number of low-correlation working condition features that affect the learning effect of the model, it is necessary to select features to reduce the training dimension. In this paper, the feature selection is combined with the LASSO selection results and the suggestions of factory experts.

LASSO was first proposed by Robert Tibshiran [28] and has been widely used in industry. It adds L1-norm regularization on the basis of simple linear regression, so that the characteristic coefficient of unimportant working conditions is reduced to zero, achieving the purpose of feature selection. The specific mathematical model of LASSO is as follows:

(1) $$\min_{\omega }\frac{1}{2}||y-{X\omega ||}_{2}^{2}+{\lambda ||\omega ||}_{1},$$

where *X* is the matrix composed of samples, *y* is the output, ω is the linear regression coefficient, and λ is the penalty function, which determines the compression degree of the regression coefficient.

Firstly, according to the industrial process, we remove the features that have no direct influence on optimal setting, such as the automatic manual switching of the flag position of the distributor valve of the preheater. Secondly, according to the LASSO feature selection method, the input feature dimension is further reduced. Finally, combined with the suggestions from factory experts, we obtain seven-dimensional features $x_{t}=\left(f_{1},f_{2},f_{3},f_{4},f_{5},f_{6},f_{7}\right)$, where $f_{i}$ represents a type of feature, as shown in Table 1.

Note that the previous optimal setting temperature of calciner is also provided as another dimension of input.

### 3.3. Data Preprocessing

**Figure 4:**
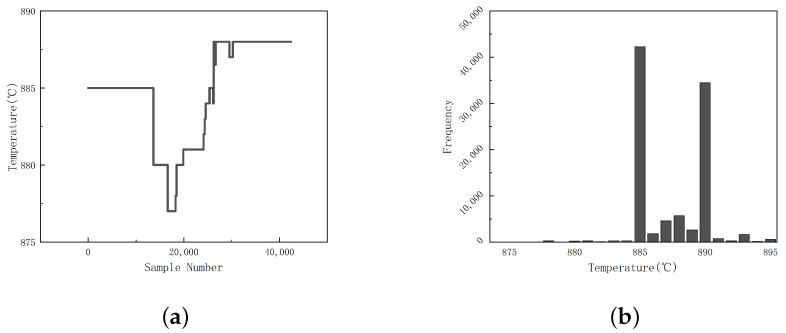
Optimal setting and frequency change chart: (**a**) setting; (**b**) frequency.

Under the condition of stable working conditions and normal equipment operation, experts do not change optimal settings frequently, which leads to a large number of data points that remain unchanged for a long time, and makes it difficult for the model to learn time series information. As shown in Figure 4a, the optimal setting on 8 July does not change in the first 10,000 data points (≈6 h), and the optimal setting interval after that is generally greater than 2000 data points (≈0.5 h).

In order to solve this problem, we slice the original dataset, and select the first 120 pieces of data when the optimal setting is changed suddenly to form a sample point. It means that the first 4 min of the working condition data when the optimal setting is changed by the experts form a sample point. This way, we can reduce the interference caused by the optimal setting remaining unchanged for a long time.

According to the expert’s experience, the optimal setting that keeps the working condition stable is between 870 and 900 °C, and the two consecutive settings generally do not exceed 5 °C. As shown in Figure 4, in the data from June to September, the optimal settings when the working condition is stable are all integers between 874 °C and 895 °C, and most of them fall in the range between 885 °C and 893 °C.

Due to the influence of factory environmental noise and equipment failure, there are interference factors such as outliers in the original dataset. We remove the abnormal data in the original dataset according to the 3σ criterion. We employ the moving average calculation method to smooth the data to avoid the influence of random error and obvious deviation in the system. Furthermore, to improve the training efficiency of the model and enhance the ability of data mining potential features, we perform the maximum–minimum value normalization to scale the data into the interval of [0, 1], as follows:

(2) $$d_{norm}=\frac{d-d_{min}}{d_{max}-d_{min}},$$

where *d* is the original data, $d_{max}$, $d_{min}$ is the maximum value and the minimum value, respectively, and $d_{norm}$ is the normalized data.

## 4. Model Implementation and Performance Evaluation

We implement the proposed prediction model for optimal temperature setting in Figure 2 based on CNN-LSTM-A with specific parameters in Table 2 using Python 3.7, Tensorflow 2.91, and Keras 2.90 framework. We run the model on a sever equipped with two 24 GB NVIDIA GeForce RTX 4090 GPUs and two Intel(R) Xeon (R) Gold 6133 @ 2.50 GHz CPUs.

CNN-LSTM-A is trained through Error Back Propagation (BP) using the Adam optimization algorithm to update the network parameters. The initial learning rate parameter is set to 0.001, and the Dropout layer retention rate parameter is set to 0.5 to avoid overfitting of the model. After many rounds of parameter optimization, the number of epochs is set to 40 and the batch size is 900. The model uses the dataset after extracting the mutation point as the training set and selects the unprocessed dataset as the test set.

The optimal setting is predicted with 120 data points as the time step, and the input time series data of the model is (120 × 8). The spatial features are extracted by one-dimensional convolution layer, and the time series information is extracted by the LSTM layer. After dropping some parameters in the Dropout layer, it is transformed into (120 × 64) vector input to the Attention layer for attention allocation. The output is compressed in the Dense layer to output the prediction for optimal setting.

### 4.1. Evaluation Metrics

According to domain experts, there is no special requirement to determine the quality of optimal setting. Therefore, the model training aims at curve fitting. In this paper, we consider common mean square error (MSE), root mean square error (RMSE), mean absolute error (MAE), mean absolute percentage error (MAPE), and determination coefficient ($R^{2}$) for performance evaluation, calculated as:

(3) $$\begin{array}{c} MSE=\frac{1}{n}\sum_{i=1}^{n}{\left(A_{i}-P_{i}\right)}^{2}, \end{array}$$

(4) $$\begin{array}{c} RMSE=\sqrt{\frac{1}{n}\sum_{i=1}^{n}{\left(A_{i}-P_{i}\right)}^{2}}, \end{array}$$

(5) $$\begin{array}{c} MAE=\frac{1}{n}\sum_{i=1}^{n}\left|A_{i}-P_{i}\right|, \end{array}$$

(6) $$\begin{array}{c} MAPE=\frac{1}{n}\sum_{i=1}^{n}\frac{|A_{i}-P_{i}|}{A_{i}}, \end{array}$$

(7) $$\begin{array}{c} R^{2}=1-\frac{\sum_{i=1}^{n}{\left(A_{i}-P_{i}\right)}^{2}}{\sum_{i=1}^{n}{\left(A_{i}-\bar{A_{i}}\right)}^{2}}, \end{array}$$

where *n* is the number of samples, $P_{i}$ is the predicted value of the sample, $A_{i}$ is the true value of the sample, and $\bar{A_{i}}$ represents the average value of the true value of the sample. Among them, the closer the first four metrics, namely Equations (3)–(6), are to 0, the better the effect is. In Equation (7), $R^{2}\in \left[0,1\right]$, it indicates the degree to which the curve is fitted, and the closer the value is to 1, the better the prediction effect of the model is. On the contrary, if the value is closer to 0, the worse the prediction effect of the model is.

### 4.2. Effects of Hyperparameters

We design three sets of comparative experiments on the hyperparameters including epoch, learning rate, and batch size. The experimental results are evaluated in terms of MAE and plotted for comparison. All experiments use the same training set in June and testing set on 8 July.

#### 4.2.1. Epochs

**Figure 5:**
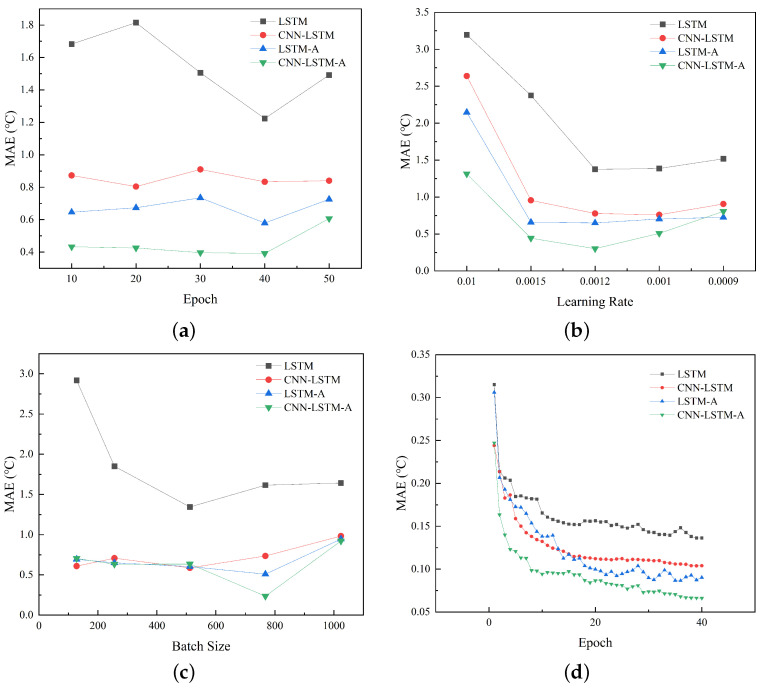
Experiment on the {epoch, learning rate, batch size} and comparison of MAE achieved by different models. (**a**) epoch; (**b**) learning rate; (**c**) batch size; (**d**) loss.

We use different epochs {10, 20, 30, 40, 50} and the same (learning rate = 0.001, batch size = 900) hyperparameters in comparative experiments. Figure 5a shows the relationship between the epochs and MAE values of four models, namely LSTM, CNN-LSTM, LSTM-A, and CNN-LSTM-A, on the test set. The CNN-LSTM-A model proposed in this paper outperforms the other three models. CNN-LSTM-A converges within 20∼30 epochs and remains unchanged within 30∼40 epochs, while the other three models take at least 40 epochs to converge, indicating that these three models require more computing resources to achieve similar results. CNN-LSTM-A starts to overfit within 40∼50 epoch, but it still maintains good performance compared with the other three models, which shows its performance superiority and adaptability to the dataset.

#### 4.2.2. Learning Rate

We analyze the influence of different learning rates {0.01, 0.0015, 0.0012, 0.001, 0.0009} on the performance of each model. The relationship between learning rate and MAE value on the test set is shown in Figure 5b. CNN-LSTM-A consistently performs better than the other three models when the learning rate is more than 0.001. With a learning rate of 0.0012, all four models achieve good results, and CNN-LSTM-A has the best performance.

#### 4.2.3. Batch Size

We analyze the impact of different batch sizes {128, 256, 512, 768, 1024} on the performance of each model. The predicted MAE values corresponding to different batch sizes on the test set are shown in Figure 5c. CNN-LSTM, LSTM-A and CNN-LSTM-A have the same performance when the batch size is less than 512, but CNN-LSTM-A has better performance when the batch size is within 512∼1024.

### 4.3. Training and Performance Evaluation

We evaluate the performance of four models, namely LSTM, CNN-LSTM, LSTM-A, and CNN-LSTM-A. The results are based on the same dataset and the optimal hyperparameter set ($h_{p}$) of each model obtained according to the evaluation metrics.

#### 4.3.1. Training Evaluation

LSTM, CNN-LSTM, LSTM-A, and CNN-LSTM-A are trained on the same training set and verification set, respectively. When the number of training epochs is 40, the MAE value of the model changes as shown in Figure 5d.

The four models converge rapidly at the initial stage of training, but the traditional LSTM model is weaker than the other three mixed models. As CNN-LSTM-A combines the ability of CNN to extract spatial features and Attention to assign weights, it achieves a faster decreasing loss and a lower error.

#### 4.3.2. Performance Evaluation

**Figure 6:**
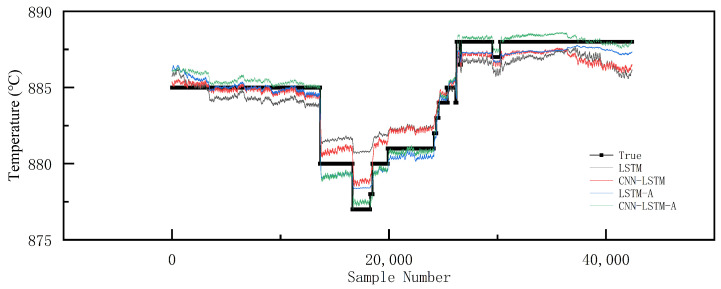
Comparison on test set.

According to Figure 6, all four models have certain effects on the fitting of the test set, but CNN-LSTM-A outperforms the other three models. According to Table 3, CNN-LSTM-A achieves lower MSE, RMSE, MAE, and MAPE than the other three models. More specifically, the MAE value of CNN-LSTM-A is about 0.04 lower than LSTM-A, 0.43 lower than CNN-LSTM, and 0.8 lower than LSTM. Moreover, CNN-LSTM-A achieves $R^{2}$ of 0.98, which indicates that CNN-LSTM-A has better capability of prediction and curve fitting and higher stability.

**Figure 7:**
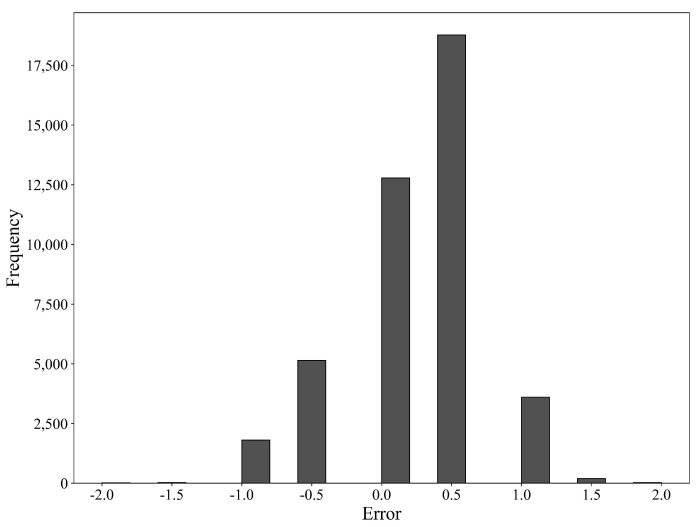
Error histogram on test set.

As illustrated in Figure 7, our model exhibits a concentration of errors between 0 and 0.5 degrees, with a maximum error not exceeding 2 degrees, aligning well with the modeling expectations. Overall, there are relatively few peaks, attributed to the low frequency and concentrated range of the original temperature settings. The prolonged stability of temperature values allows the model to learn such features, resulting in outcomes that fluctuate within a small range after prediction, meeting the practical production requirements.

The CNN-LSTM-A source domain model with the best training effect is saved, and the online datasets in November and December are used as the target domain to import the model for prediction. The prediction results of different models are provided in Table 4 for comparison.

**Figure 8:**
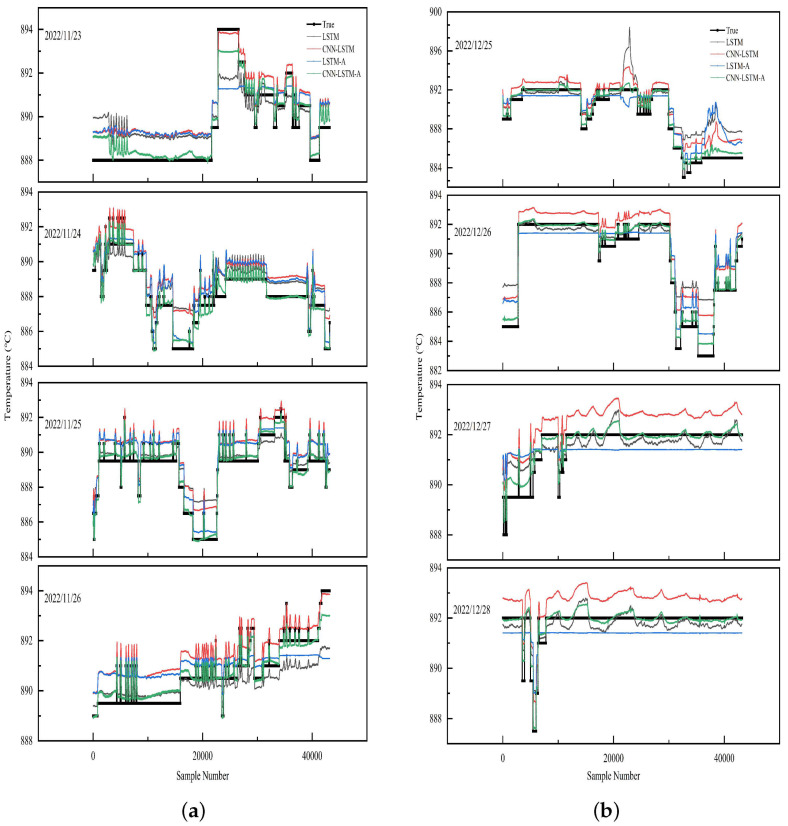
Comparison chart of optimal setting prediction results of multiple models: (**a**) 11/23∼11/26 comparison; (**b**) 12/25∼12/28 comparison.

Figure 8a shows that CNN-LSTM-A has the best prediction performance in the online data test from 11.23 to 11.26. According to Table 4, the MAE value of CNN-LSTM-A model is less than 0.4 °C, and the $R^{2}$ value reaches 0.96, which fits the optimal setting by experts very well. However, the MAE values of CNN-LSTM and LSTM-A, which perform well in the training set, increase compared with the training phase, and their $R^{2}$ values are less than 0.8.

To better illustrate the advantages of CNN-LSTM-A, we also test on the online dataset of 12.25∼12.28. Figure 8b and Table 5 show the prediction results of different models. CNN-LSTM-A is still the best, but the performance of LSTM-A has changed: its $R^{2}$ value reaches 0.86 with good fitting effect, which shows that LSTM-A is not stable. However, the other two models cannot predict the optimal setting value well, and their MAE value is greater than 1 °C. These results show that CNN-LSTM-A has the best overall performance among all models in comparison.

**Figure 9:**
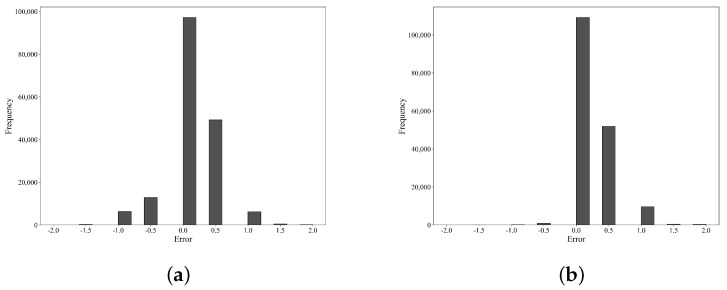
Error histogram in November and December: (**a**) November; (**b**) December.

In Figure 9a,b, we observe that during the actual tests in November and December, the distribution of errors is concentrated in the range of 0–0.5 ${}^{{}^{\circ}}$C. This indicates that the model aligns well with the operator’s experience regarding changes in set values. The distribution shows fewer peaks, suggesting that the model maintains stability after predictions and exhibits excellent capability for correcting exceptional situations.

## 5. Conclusions

To maintain the normal operation of decomposition furnaces and other equipment in cement plants and ensure the output of high-quality cement products, we proposed an optimal setting prediction model, CNN-LSTM-A. This model uses CNN for spatial feature extraction, LSTM for time series information extraction, and attention mechanism for weight distribution optimization to improve prediction accuracy.

To illustrate the superiority of this model, we analyze the prediction effect of different models including LSTM, CNN-LSTM, and LSTM-A in terms of MSE, RMSE, MAE, MAPE, and $R^{2}$ under the optimal hyperparameter set ($h_{p}$). We trained these models using the source domain data and tested them on the target domain data. The results show that the CNN-LSTM-A can accurately predict the optimal setting value with an error less than 0.4. It provides a promising solution for optimal setting prediction and has potentials for production use in cement plants.

## Figures and Tables

**Table 1:** Seven selected features.

Number	Features
$f_{1}$	the $NO_{x}$ concentration in the kiln tail smoke chamber
$f_{2}$	the head coal feedback
$f_{3}$	the kiln tail smoke chamber temperature
$f_{4}$	the outlet temperature of the calciner
$f_{5}$	the kiln feed feedback
$f_{6}$	the secondary air temperature
$f_{7}$	the rotary kiln current

**Table 2:** Neural network structure.

Number	Structure
1	Conv1D (4 filters)
2	LSTM (64 units)
3	Dropout (0.5)
4	Attention (64 units)
5	Dense (1 neuron, RELU activation)

**Table 3:** Performance comparison of different models for optimal setting forecast on the same test set.

Model	MSE	RMSE	MAE	MAPE	$R^{2}$
LSTM	1.894464	1.376395	1.166849	0.001320	0.81565
CNN-LSTM	0.921729	0.960067	0.791589	0.000895	0.910306
LSTM-A	0.283533	0.532478	0.402836	0.000456	0.972409
**CNN-LSTM-A**	**0.261575**	**0.501311**	**0.364859**	**0.000426**	**0.984815**

**Table 4:** Performance comparison of different models for optimal setting forecast during the period of 11.23∼11.26.

Model	MSE	RMSE	MAE	MAPE	$R^{2}$
LSTM	1.331596	1.153948	0.964812	0.001084	0.713240
CNN-LSTM	1.061954	1.030512	0.918858	0.001033	0.771308
LSTM-A	1.260732	1.122823	0.982102	0.001103	0.728501
**CNN-LSTM-A**	**0.172914**	**0.415829**	**0.302435**	**0.000340**	**0.962763**

**Table 5:** Performance comparison of different models for optimal setting forecast during the period of 12.25∼12.28.

Model	MSE	RMSE	MAE	MAPE	$R^{2}$
LSTM	3.191160	1.786382	1.216378	0.001372	0.702751
CNN-LSTM	2.290155	1.513326	1.334463	0.001503	0.786677
LSTM-A	1.466172	1.210856	1.007284	0.001134	0.863430
**CNN-LSTM-A**	**0.234398**	**0.484146**	**0.339946**	**0.000383**	**0.978166**

## Data Availability

The data presented in this study are available on request from the corresponding author. The data are not publicly available due to privacy and confidentiality concerns.
